# Aberrant regulation of RANKL/OPG in women at high risk of developing breast cancer

**DOI:** 10.18632/oncotarget.14013

**Published:** 2016-12-18

**Authors:** Stefan Kiechl, Daniel Schramek, Martin Widschwendter, Evangelia-Ourania Fourkala, Alexey Zaikin, Allison Jones, Bernadette Jaeger, Brigitte Rack, Wolfgang Janni, Christoph Scholz, Johann Willeit, Siegfried Weger, Agnes Mayr, Andrew Teschendorff, Adam Rosenthal, Lindsay Fraser, Susan Philpott, Louis Dubeau, Mohammed Keshtgar, Rebecca Roylance, Ian J. Jacobs, Usha Menon, Georg Schett, Josef M. Penninger

**Affiliations:** ^1^ Department of Neurology, Medical University of Innsbruck, Innsbruck, Austria; ^2^ IMBA, Institute of Molecular Biotechnology of the Austrian Academy of Sciences, Vienna, Austria; ^3^ The Lunenfeld-Tanenbaum Research Institute, Mount Sinai Hospital, Toronto, Ontario, Canada; ^4^ Department of Women’s Cancer, EGA Institute of Women’s Health, University College London, London, United Kingdom; ^5^ Department of Mathematics, University College London, London, United Kingdom; ^6^ Department of Gynecology and Obstetrics, University Duesseldorf, Duesseldorf, Germany; ^7^ Heinrich-Heine-University Dusseldorf, Dusseldorf, Germany; ^8^ Department of Internal Medicine, Bruneck Hospital, Bruneck, Italy; ^9^ Statistical Genomics Group, Paul O’Gorman Building, UCL Cancer Institute, University College London, London, United Kingdom; ^10^ Barts Cancer Institute CR UK Centre of Excellence, Queen Mary University of London, Charterhouse Square, London, United Kingdom; ^11^ Department of Pathology, USC/Norris Comprehensive Cancer Center, Keck School of Medicine, University of Southern California, Los Angeles, California, USA; ^12^ Department of Surgery, Royal Free and University College London Medical School, London, United Kingdom; ^13^ UNSW Australia, Sydney, New South Wales, Australia; ^14^ Department of Internal Medicine 3, University of Erlangen-Nuremberg, Erlangen, Germany

**Keywords:** breast cancer, RANKL/RANK, Gerotarget

## Abstract

Breast cancer is the most common female cancer, affecting approximately one in eight women during their lifetime in North America and Europe. Receptor Activator of NF-kB Ligand (RANKL), its receptor RANK and the natural antagonist osteoprotegerin (OPG) are essential regulators of bone resorption. We have initially shown that RANKL/RANK are essential for hormone-driven mammary epithelial proliferation in pregnancy and RANKL/RANK have been implicated in mammary stem cell biology. Using genetic mouse-models, we and others identified the RANKL/RANK system as a key regulator of sex hormone, *BRCA1*-mutation, and oncogene-driven breast cancer and we proposed that RANKL/RANK might be involved in the initiation of breast tumors. We now report that in postmenopausal women without known genetic predisposition, high RANKL and progesterone serum levels stratify a subpopulation of women at high risk of developing breast cancer 12-24 months before diagnosis (5.33-fold risk, 95%CI 1.5-25.4; *P=*0.02). In women with established breast cancer, we demonstrate that RANKL/OPG ratios change dependent on the presence of circulating tumor cells (CTCs). Finally, we show in a prospective human breast cancer cohort that alterations in RANKL/OPG ratios are significantly associated with breast cancer manifestation. These data indicate that the RANKL/RANK/OPG system is deregulated in post-menopausal women at high risk for breast cancer and in women with circulating tumor cells. Thus, serum levels of RANKL/OPG are potentially indicative of predisposition and progression of breast cancer in humans. Advancement of our findings towards clinical application awaits prior validation in independent patient cohorts.

## INTRODUCTION

RANKL (receptor activator of NF-kB ligand), its receptor RANK, and the decoy receptor osteoprotegerin (OPG) are essential for the development and activation of osteoclasts [1, 2]. Based on these findings, RANKL inhibition with a fully human, blocking monoclonal antibody (Denosumab) has been developed as a novel and rational therapy against osteoporosis and skeletal related events in cancer patients [3–7]. RANKL/RANK also control lymph node organogenesis and the development of thymic medullary epithelial cells [1, 8, 9]. In addition, we have previously shown that the RANKL/RANK system is essential for the formation of a lactating mammary gland during pregnancy [10, 11] indicating that RANKL/RANK play a role in normal mammary gland biology and hormone-driven epithelial proliferation. Indeed, using genetic mouse-models, our group and Gonzalez-Suarez *et al*. have identified the RANKL/RANK system as a key regulator of hormone (progestin) and oncogene (Neu)-driven breast cancer [12, 13]. Moreover, we [14] and others [15] have recently reported that RANKL/RANK also control breast cancer development in scenarios of BRCA1 mutations. Mechanistically, RANKL/RANK promote the proliferation of human and mouse mammary gland epithelial cells, protect these cells from apoptotic cell death after DNA damage, control tumor stem cell renewal, and might have a role in basic mammary stem cell biology [12–18]. Furthermore, RANKL/RANK have also been implicated in metastases [12, 13, 19, 20].

Breast cancer is the most common female cancer, affecting approximately one in eight women during their life-time in North America and Europe [21, 22]. Identifying a biomarker which serves as both an indicator for breast cancer risk and at the same time a possible target for prevention is one of the most important yet unsolved needs. RANKL is expressed in primary breast cancers in patients and human breast cancer cells lines [14–19, 23] and, in mouse studies, the RANKL/RANK system is an important molecular link between progestins and oncogene-driven epithelial carcinogenesis [12, 13]. We therefore hypothesized that serum levels of RANKL and OPG could serve as markers for breast cancer risk providing a molecular rationale for future breast cancer prevention.

## RESULTS

### High serum levels of RANKL and progesterone stratify a subpopulation of women at increased risk of developing breast cancer

In order to test whether deregulation of the RANKL/OPG system is associated with breast cancer in women without known genetic predispositions, we analyzed serum levels in postmenopausal women participating in the prospective UKCTOCS (UK Collaborative Trial of Ovarian Cancer Screening) study [24, 25] (Table 1). This cohort of women provided a unique opportunity to study changes in serum levels of soluble RANKL and OPG well in advance of breast cancer manifestation. A total of 278 samples from postmenopausal women were analyzed: 40 women donated a serum sample between 5 and 12 months prior to breast cancer diagnosis (median age at sample taken 64.84), 58 women provided sera between 12 and 24 months prior to breast cancer diagnosis (median age at sample taken 60.49 years) and 180 women, who did not develop breast cancer during their follow up (median age at sample taken 62.94 years), served as controls.

**Table 1:** Clinicopathological features of women who developed invasive breast cancer within the UKCTOCS trial 5 to 24 months after they provided their serum sample

Clinicopathological Characteristics	Breast Cancer Diagnosis after	
	5-12mo	12-24mo
	(*n*= 40)	(*n*= 58)
**Histology Classification**		
IDC	27	48
ILC	9	9
ITC	1	0
Other	1	1
Unknown	2	0
**Stage (TNM)**		
1	21	29
2	3	14
3	1	2
Unknown	15	13
**Grade**		
I	9	9
II	24	29
III	7	20
**Hormone receptor status**		
ER positive	40	58
PR negative	8	22
PR positive	20	8
PR unknown	12	28
**HER2**		
HER2 negative	15	17
HER2 positive	4	4
Unknown	21	37
**Nodal Status**		
Positive	7	13
Negative	33	45

* IDC, invasive ductal cancer; ILC, invasive lobular cancer; ITC, invasive tubular cancer.

**Figure 1:**
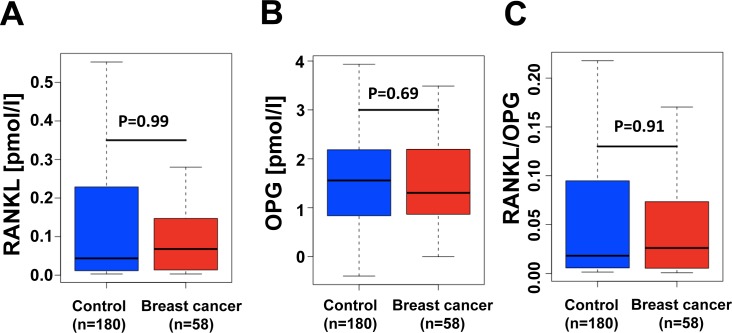
RANKL/OPG ratios are not changed in women that develop breast cancer within 12-24 month after serum sampling **A.-C.** Analysis of individual RANKL and OPG levels in prospectively collected serum samples from UKCTOCS (UK Collaborative Trial of Ovarian Cancer Screening) from 180 healthy postmenopausal women who did not develop breast cancer during their follow up and 58 healthy age-matched women who did develop estrogen receptor positive breast cancer 12-24 months after their serum was collected. Box plots of RANKL **A.**, OPG **B.** levels as well as RANKL-to-OPG ratios C. are shown. There were no significant differences (Mann Whitney U test). Box plots indicate median ratio levels and inter-quartile ranges.

**Figure 2:**
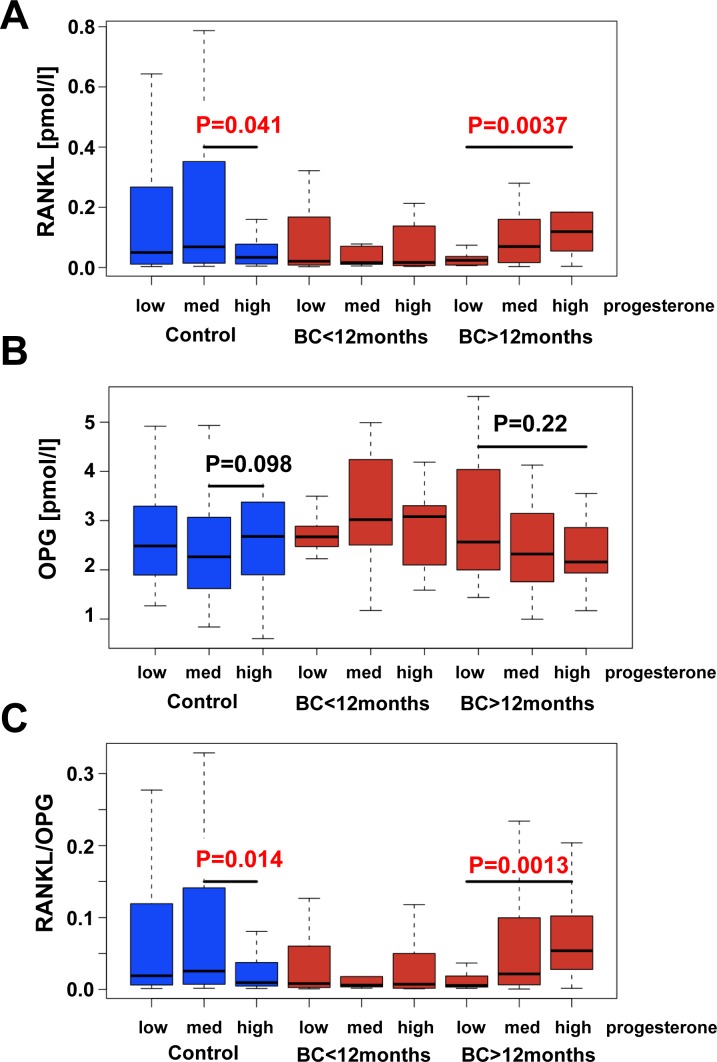
RANKL/OPG serum levels in human breast cancer patients Analysis of RANKL **A.**, OPG **B.** and the ratio RANKL/OPG **C.** in relation to progesterone levels in prospectively collected UKCTOCS serum samples from 180 healthy postmenopausal women who did not develop breast cancer during their follow up and 40 healthy age-matched women who developed estrogen receptor positive breast cancer 5-12 months after their serum was collected as well as 58 healthy age-matched women who did develop ER-positive breast cancer 12-24 months after their serum was collected. Women were grouped according to their serum progesterone levels. Subjects were stratified into breast cancer patients and controls due to self reporting and histological examination. We have tested differences in RANKL, OPG and RANKL/OPG within each group between low *versus* medium and medium *versus* high progesterone within each group using the Mann Whitney U test and displayed only the significant p-values (*p* < 0.05).

We did not observe significant differences in RANKL, OPG or RANKL-to-OPG ratios in controls and women who developed breast cancer 12-24 months after serum sampling (Figure 1A-1C). Since we have previously shown that RANKL mediates progesterone driven mammary epithelial proliferation [12] and the RANKL/RANK system is a key mediator of progestin-driven breast cancer in mouse models [12, 13], we explored a potential connection between progesterone and RANKL/OPG levels in humans. Intriguingly, we observed an increase of RANKL serum levels with increasing progesterone levels in women who developed breast cancer 12-24 months after sample collection (Figure 2A) and the opposite trend in controls. In the same cohort, we also observed a non-significant negative correlation of progesterone with OPG serum levels (Figure 2B) resulting in a significant alteration in the RANKL/OPG ratio among women with high serum progesterone (Figure 2C).

To assess whether high progesterone and high RANKL indeed define a subgroup of women with increased risk of developing breast cancer, we classified all women - cases and controls considered together - into tertiles according to their serum progesterone levels. Within each group women were classified into low, medium or high RANKL, OPG and RANKL/OPG based on controls within the corresponding progesterone group. Using logistic regression women with high progesterone and high RANKL exhibited a 4.8 (95% CI 1.3 - 22.8; *p* = 0.0269) fold risk (Table 2). OPG levels alone did not modify the risk (Table 3). Importantly, women within the high progesterone and high serum RANKL/OPG ratio group carried a 5.3 (95% CI 1.5 - 25.4, *p* = 0.0169) fold risk to develop breast cancer 12-24 months after diagnosis (Table 4). We also grouped the women into RANKL/OPG tertiles across all controls (not only within a given progesterone tertile) and again women within the high progesterone/high RANKL/OPG group had a 4.2 (95% CI 1.3 - 16.0, *p* = 0.02) fold risk for developing breast cancer. Thus, high serum levels of RANKL and high serum progesterone stratify a subpopulation of postmenopausal women without known genetic predisposition at high risk of developing breast cancer 12-24 months before diagnosis.

**Table 2:** Association of serum progesterone and RANKL with risk of breast cancer

Progesterone LOW (range 0.03 - 0.19 ng/ml)*																
RANKL [pmol/l]			Controls		Breast Cancer < 12 months						Breast Cancer > 12 months					
Tertile^$^	Range		*N* (%)		*N* (%)		OR (95% CI)^¶^			*P*-value	*N* (%)		OR (95% CI)^¶^			*P*-value
1st	0.003	0.0193	21	33.9	7	50.0	1(ref)				6	42.9	1(ref)			
2nd	0.0193	0.179	20	32.3	4	28.6	0.60	0.1	2.3	0.47	8	57.1	1.40	0.4	4.9	0.59
3rd	0.179	3.716	21	33.9	3	21.4	0.43	0.1	1.8	0.26	0	0.0	0.00	NA		0.99
Progesterone MEDIUM (range 0.19 - 0.31 ng/ml)*																
**RANKL [pmol/l]**			**Controls**		**Breast Cancer < 12 months**						**Breast Cancer > 12 months**					
Tertile^$^	Range		*N* (%)		*N* (%)		OR (95% CI)^¶^			*P*-value	N (%)		OR (95% CI)^¶^			*P*-value
1st	0.004	0.0255	18	32.1	6	60.0	1(ref)				8	34.8	1(ref)			
2nd	0.0255	0.1745	19	33.9	3	30.0	0.47	0.1	2.1	0.34	9	39.1	1.07	0.3	3.4	0.91
3rd	0.1745	1.5235	19	33.9	1	10.0	0.16	0.0	1.0	0.10	6	26.1	0.71	0.2	2.4	0.59
Progesterone HIGH (0.31 - 12.68 ng/ml)*																
**RANKL [pmol/l]**			**Controls**		**Breast Cancer < 12 months**						**Breast Cancer > 12 months**xs					
Tertile^$^	Range		*N* (%)		*N* (%)		OR (95% CI)^¶^			*P*-value	N (%)		OR (95% CI)^¶^			*P*-value
1st	0.0045	0.0135	20	32.3	5	31.3	1(ref)				3	14.3	1(ref)			
2nd	0.0135	0.0675	21	33.9	5	31.3	0.95	0.2	3.9	0.94	3	14.3	0.95	0.2	5.7	0.96
3rd	0.0675	1.2315	21	33.9	6	37.5	1.14	0.3	4.5	0.84	15	71.4	4.76	1.3	22.8	0.03

* All women - cases and controls considered together – were classified into tertiles according to their serum progesterone levels.

$ Within each group, women were classified into low, medium or high RANKL based on controls within the corresponding progesterone group.

¶ Using logistic regression odd ratios (ORs) and 95% Confidence Intervals (CI) have been calculated using the lowest RANKL group as a reference. Cases have been considered separately according to the time to diagnosis (more than one year or less).

**Table 3:** Association of serum progesterone and OPG with risk of breast cancer

Progesterone LOW (range 0.03 - 0.19 ng/ml)*																
OPG [pmol/l]			Controls		Breast Cancer < 12 months						Breast Cancer > 12 months					
Tertile^$^	Range		*N* (%)		*N* (%)		OR (95% CI)¶			*P*-value	*N* (%)		OR (95% CI)¶			*P*-value
1st	1.2685	2.0267	21	33.9	1	7.1	0.33	0.0	2.8	0.36	6	42.9	0.86	0.2	3.0	0.81
2nd	2.0267	2.9685	20	32.3	10	71.4	3.50	0.9	17.3	0.09	1	7.1	0.15	0.0	1.0	0.09
3rd	2.9685	4.917	21	33.9	3	21.4	1(ref)				7	50.0	1(ref)			
Progesterone MEDIUM (range 0.19 - 0.31 ng/ml)*																
**OPG [pmol/l]**			**Controls**		**Breast Cancer < 12 months**						**Breast Cancer > 12 months**					
Tertile^$^	Range		*N* (%)		*N* (%)		OR (95% CI)¶			*P*-value	*N* (%)		OR (95% CI)¶			*P*-value
1st	0.8385	1.8617	19	33.9	2	20.0	0.40	0.1	2.1	0.31	7	30.4	0.88	0.3	2.9	0.83
2nd	1.8617	2.7008	18	32.1	3	30.0	0.63	0.1	3.0	0.57	8	34.8	1.06	0.3	3.5	0.93
3rd	2.7008	4.9335	19	33.9	5	50.0	1(ref)				8	34.8	1(ref)			
Progesterone HIGH (0.31 - 12.68 ng/ml)*																
**OPG [pmol/l]**			**Controls**		**Breast Cancer < 12 months**						**Breast Cancer > 12 months**					
Tertile^$^	Range		*N* (%)		*N* (%)		OR (95% CI)¶			*P*-value	*N* (%)		OR (95% CI)¶			*P*-value
1st	0.6015	2.0855	21	33.9	4	25.0	0.40	0.1	1.4	0.17	10	47.6	2.00	0.6	7.4	0.27
2nd	2.0855	2.9933	20	32.3	2	12.5	0.21	0.0	0.9	0.06	6	28.6	1.26	0.3	5.0	0.73
3rd	2.9933	9.9125	21	33.9	10	62.5	1(ref)				5	23.8	1(ref)			

* All women - cases and controls considered together – were classified into tertiles according to their serum progesterone levels.

$ Within each group, women were classified into low, medium or high OPG based on controls within the corresponding progesterone group.

¶ Using logistic regression odd ratios (ORs) and 95% Confidence Intervals (CI) have been calculated using the highest OPG group as a reference. Cases have been considered separately according to the time to diagnosis (more than one year or less).

**Table 4:** Association of serum progesterone and RANKL/OPG ratio with risk of breast cancer

Progesterone LOW (range 0.03 - 0.19 ng/ml)*																	
RANKL/OPG ratio				Controls		Breast Cancer < 12 months						Breast Cancer > 12 months					
Tertile^$^		Range		*N* (%)		*N* (%)		OR (95% CI)¶			*P*-value	*N* (%)		OR (95% CI)¶			*P*-value
1st		0.0014	0.0075	21	33.9	7	50.0	1(ref)				9	64.3	1(ref)			
2nd		0.0075	0.0644	20	32.3	4	28.6	0.60	0.1	2.3	0.47	5	35.7	0.58	0.2	2.0	0.40
3rd		0.0644	2.0472	21	33.9	3	21.4	0.43	0.1	1.8	0.26	0	0.0	0.00	NA	NA	1.0
Progesterone MEDIUM (range 0.19 - 0.31 ng/ml)*																	
**RANKL/OPG ratio**				**Controls**		**Breast Cancer < 12 months**						**Breast Cancer > 12 months**					
Tertile^$^	Range			*N* (%)		*N* (%)		OR (95% CI)¶			*P*-value	*N* (%)		OR (95% CI)¶			*P*-value
1st	0.0017		0.0154	19	33.9	6	60.0	1(ref)				8	34.8	1(ref)			
2nd	0.0154		0.091	18	32.1	4	40.0	0.70	0.2	2.9	0.63	9	39.1	1.19	0.4	3.8	0.77
3rd	0.091		0.4605	19	33.9	0	0.0	0.00	NA		0.99	6	26.1	0.75	0.2	2.6	0.65
Progesterone HIGH (0.31 - 12.68 ng/ml)*																	
**RANKL/OPG ratio**				**Controls**		**Breast Cancer < 12 months**						**Breast Cancer > 12 months**					
Tertile^$^	Range			*N* (%)		*N* (%)		OR (95% CI)¶			*P*-value	*N* (%)		OR (95% CI)¶			*P*-value
1st	0.0015		0.0057	21	33.9	6	37.5	1(ref)				3	14.3	1(ref)			
2nd	0.0057		0.0245	20	32.3	4	25.0	0.70	0.2	2.8	0.62	2	9.5	0.70	0.1	4.7	0.71
3rd	0.0245		0.4672	21	33.9	6	37.5	1.00	0.3	3.7	1.00	16	76.2	5.33	1.5	25.4	0.02

* All women - cases and controls considered together – were classified into tertiles according to their serum progesterone levels.

$ Within each progesterone tertile, women were classified into low, medium or high RANKL/OPG based on controls within the corresponding progesterone group.

¶ Using logistic regression odd ratios (ORs) and 95% Confidence Intervals (CI) have been calculated using the lowest RANKL/OPG group as a reference. Cases have been considered separately according to the time to diagnosis (more than one year or less).

### RANKL/OPG levels in females close to onset of breast cancer diagnosis and cancer patients with circulating tumor cells

**Figure 3:**
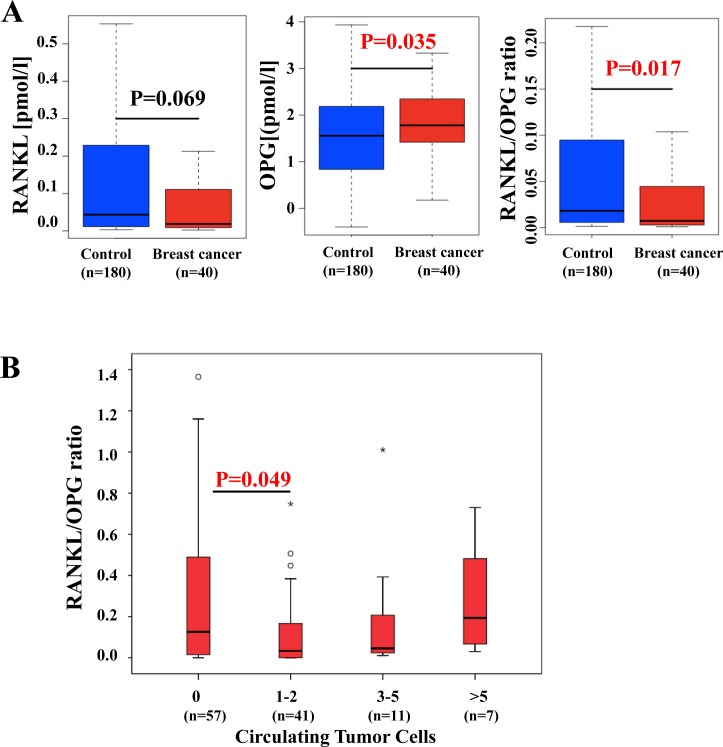
Alterations in RANKL/OPG levels in females close to onset and after manifestation of breast cancer **A.** Analysis of OPG, RANKL, and RANKL/OPG ratios in prospectively collected UKCTOCS serum samples from 180 healthy postmenopausal women who did not develop breast cancer during their follow up (control) and 40 healthy age-matched women who developed ER positive breast cancer 5-12 months after their serum was collected. P values were calculated using a Mann Whitney U test. **B.** Analysis of RANKL/OPG ratios in 116 women in the SUCCESS trial *after resection* of the local ER positive breast cancer and *before start of systemic treatment* based on the numbers of CTCs. P values were calculated using the Mann Whitney U test. Abbreviations: CTC, circulating tumor cell; ER, estrogen receptor; OPG, osteoprotegerin; RANKL, Receptor Activator of NF-kB ligand.

We next analyzed the group of UKCTOCS participants who developed breast cancer within 12 months (BC < 12 months). However, among this cohort high progesterone/high RANKL levels were not associated with an increased incidence of breast cancer (Figure 2, Table 4). Intriguingly, we rather observed a progesterone-independent reduction in RANKL and increase in OPG serum levels with breast cancer manifestation resulting in significant decrease in the RANKL-to-OPG ratio compared to UKCTOCS participants who never developed breast cancer (Figure 3A). These results indicate that RANKL levels drop whereas OPG levels increase in women close to clinical manifestation of breast tumors. One possible explanation for this phenomenon could be that some women already harbor subclinical disseminated tumor cells that could lead to the observed alterations in serum RANKL/OPG.

To test this hypothesis, we analysed serum RANKL/OPG in women with breast cancer diagnosis but in the absence of a tumor in the breast - a similar scenario as in the UKCTOCS cohort close to breast cancer diagnosis. We analyzed 116 ER positive breast cancer patients from the SUCCESS trial after surgery and before systemic therapy (Table 5). Figure 3B demonstrates serum RANKL/OPG levels stratified to the number of circulating tumor cells (CTCs) identified in the corresponding blood sample [26, 27]. Intriguingly, women with a small number of CTCs (1-2 CTCs) exhibited a significantly reduced serum RANKL/OPG ratio compared to women without detectable CTCs (Figure 3B). Women with a 3-5 CTCs also exhibited reduced serum RANKL/OPG ratios, though this reduction did not reach statistical significance. Of note, the serum RANKL/OPG ratio tends to increase again with an increase in CTCs, albeit the numbers of cases is too small to allow for a firm conclusion. Thus, women with a very low number of CTCs in their blood exhibit a reduced RANKL/OPG ratio suggesting that the alterations we find up to one year in advance of a clinical breast cancer indeed correlate with low numbers of disseminated breast cancer cells.

**Table 5:** Clinicopathological features of the 116 ER positive SUCCESS patients

Clinicopathological Characteristics	
Histology Classification	No
IDC	81
ILC	27
Other	8
**Stage (T)**	
1	41
2	62
3	12
4	1
**Grade**	
I	3
II	76
III	37
**HER2**	
positive	20
negative	96
**CTC**	
Present	59
Absent	57

Abbreviations: CTC, circulating tumor cells; ER, estrogen receptor; HER2, human epidermal growth factor receptor 2; IDC invasive, ductal cancer; ILC, invasive lobular cancer.

### Confirmation of altered RANKL/OPG ratios in an independent prospective breast cancer cohort

**Figure 4:**
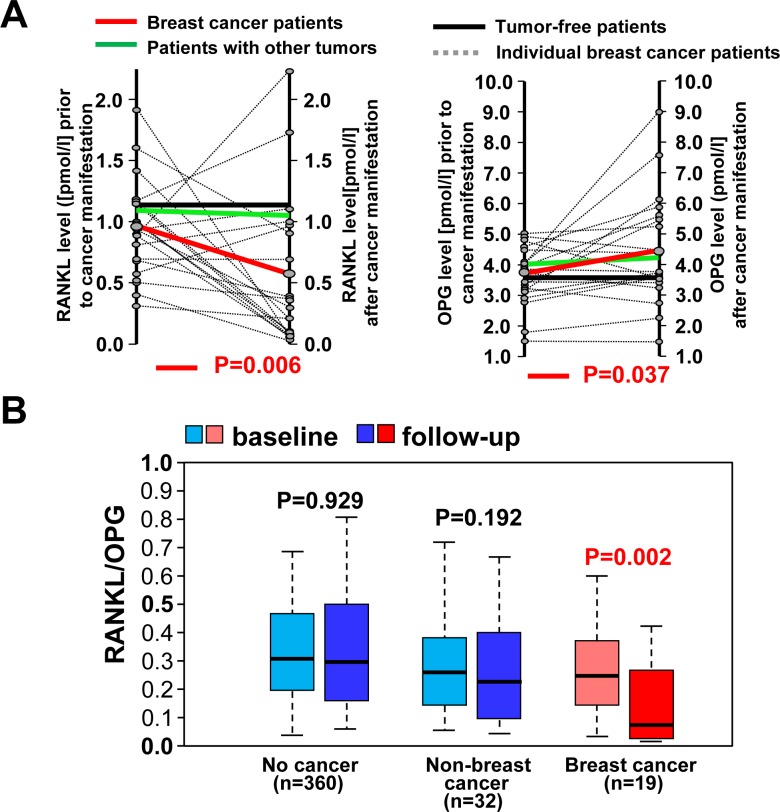
Alterations in RANKL/RANKL levels in females close to onset and after breast cancer **A.** Individual changes in serum levels of OPG and soluble RANKL in 19 female subjects from our longitudinal Bruneck cohort before and after manifestations of breast cancer (dotted black lines). Mean changes are shown for subjects with incident breast cancer (red lines), other types of new-onset cancer (*n* = 32; green lines) and women free of neoplastic disease during the 15 year follow-up (*n* = 360; black lines). **B.** Box plots of RANKL-to-OPG ratios assessed prior to and after cancer manifestation in women from the prospective Bruneck study indicate median ratio levels and inter-quartile ranges. In individuals who remained free of cancer, ratios given are those assessed at the time intervals corresponding to those in cancer patients.

**Figure 5:**
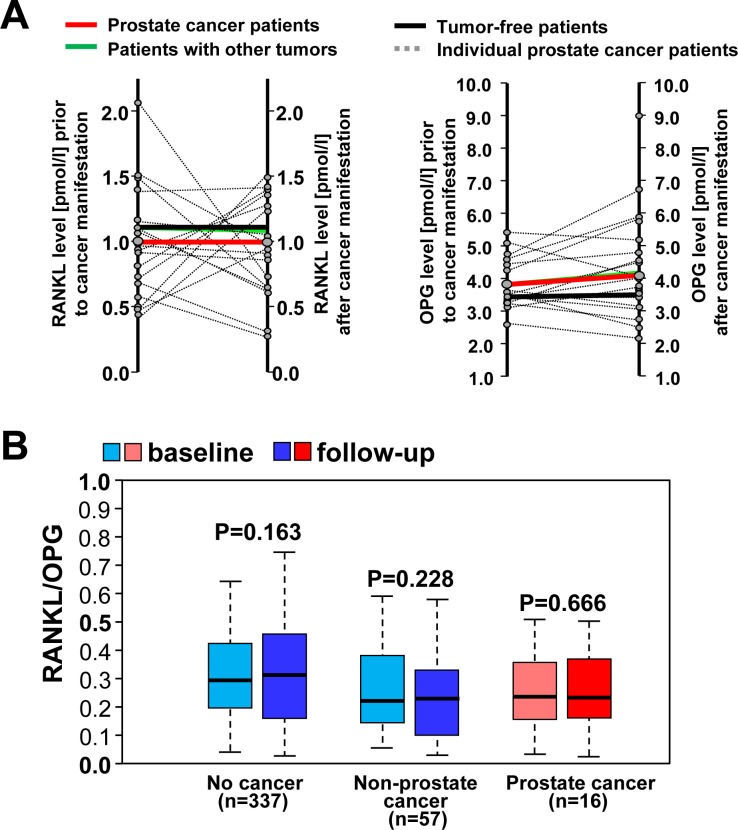
No changes in RANKL/OPG ratios in man that develop prostate cancer or females with non-breast cancer **A.** Individual changes in serum levels of osteoprotegerin (OPG) and soluble (s) RANKL in 16 male subjects from our longitudinal Bruneck cohort before and after manifestations of prostate cancer (dotted black lines). Mean changes are shown for subjects with incident prostate cancer (red lines), other types of new-onset cancer (*n* = 57; green lines) and men free of neoplastic disease during the 15 year follow-up (*n* = 337; black lines). **B.** Box plots of RANKL-to-OPG ratios indicate median ratio levels and inter-quartile ranges. Data were assessed prior to and after prostate cancer manifestation, males with other types of new-onset cancer and men free of neoplastic disease during the 15 year follow-up. For individuals who remained free of cancer, ratios given are those assessed at the time intervals corresponding to those in cancer patients.

To substantiate these data in another entirely independent cohort, we determined RANKL and OPG serum levels of participants from the Bruneck study [28, 29], that allowed us to analyse matched patient samples before and after cancer manifestation. Of 821 subjects with two or more sequential measurements of RANKL and OPG, 697 subjects remained free of cancer during the 15-year follow-up, 19 women developed breast cancer, 16 men developed prostate cancer, and 89 other cancer types (all malignancies except for squamous-cell skin cancer). In line with the UKCTOCS study, levels of RANKL declined with the manifestation of breast cancer (*p* = 0.006; paired *t*-test), whereas RANKL serum concentrations were not altered in subjects with other types of new-onset cancer and those remaining free of neoplastic disease (Figure 4A). In parallel, OPG levels rose with the diagnosis of breast cancer (*p* = 0.037; paired *t*-test), whereas no such increase was observed in patients with other types of cancer or subjects free of neoplastic disease (Figure 4A). As a consequence, the RANKL-to-OPG ratio significantly decreased in individuals with new-onset breast cancer (Figure 4B). In males, no significant differences in OPG and RANKL levels were found in cancer-free individuals or those with new onset prostate cancer, which is also related to sex hormones, or other types of cancer (Figure 5A, 5B). Our data in two different human prospective breast cancer cohorts indicate that alterations in RANKL/OPG ratios are significantly associated with breast cancer manifestation.

## DISCUSSION

We and other groups have previously demonstrated a central role for the osteoclastogenic molecule RANKL in breast cancer development using genetic mouse models [12–15]. Here we show that increased progesterone and RANKL serum levels stratify a subgroup of postmenopausal women without known genetic predispositions that exhibit a ~5 fold increased risk of developing breast cancer 12-24 months before cancer diagnosis. This indicates that either increased free RANKL or high RANKL and high progesterone serum levels, as seen in the case of our postmenopausal UK cohort, could be useful biomarkers to predict future breast cancer more than one year in advance of breast cancer diagnosis. These data are in line with our genetic mouse models that the RANKL/RANK/OPG system drives the initiation and progression of breast cancer [12–15]. Our findings that an increased RANKL/OPG ratio > 12 months prior to breast cancer development is consistent with findings from meta-analysis by Key *et al*. that showed that sex steroid hormone levels well in advance of breast cancer diagnosis are more significantly associated with breast cancer risk compared with hormone levels analyzed closer to diagnosis [30].

When we analyzed the serum of postmenopausal women shortly before breast cancer diagnosis or matched sera from women before and after clinical manifest breast cancer, we observed reduced RANKL and increased OPG serum levels. A similar phenomenon was shown for serum RANKL levels and osteoporosis: low serum RANKL levels are associated with a 10-fold higher risk of non-traumatic fractures in postmenopausal women [31]. Moreover, increased OPG is associated with enhanced bone loss in postmenopausal women not on hormone replacement therapy [32] and increased OPG levels have been observed in patients with bone metastasis [33]. The mechanisms of these serum changes in breast cancer need to be further investigated and might reflect compensatory mechanisms against developing microtumors and/or redistribution/sequestration of RANKL/OPG within different body compartments. Although women in our study do not have clinical manifest bone metastasis, it has been demonstrated that tumor cells can disseminate systemically from earliest epithelial alterations in transgenic mice and even from very early breast cancers in women and that these disseminated tumor cells can form micrometastasis in bone marrow and lungs [34]. Likewise, it is well established that breast cancer cells interfere with osteoblasts (being a major source for RANKL) and induce osteoblast necrosis and apoptosis [35]. Hence the effect we see on RANKL and the RANKL/OPG ratio in women who get clinical manifest breast cancer close to serum collection may be triggered by an effect of epithelial/tumor cells altering RANKL/OPG in the bone marrow. Of note, recently it has been reported that blocking of RANKL reduces the reoccurrence of breast cancer in women receiving adjuvant Tamoxifen therapy [36].

One key issue for the management of cancer is to accurately predict the risk in order to triage women into screening or preventive strategies. In breast cancer, population-based mammography screening is currently used to identify women with early breast cancer. Our data suggest that the apparent alterations in RANKL/OPG in postmenopausal women with high progesterone levels could be used as a future biomarker to identify a subgroup of women at high risk of breast cancer. Moreover, it has been recently shown that women with high genetic breast cancer risk due to *BRCA1* mutations appear to be exposed to increased RANKL [37]. Our data also indicate that serum RANKL/OPG ratios correlate with CTCs and that a low number of CTCs - even in the absence of a local breast cancer - is associated with a reduced serum RANKL/OPG ratio. The mechanism of this phenomenon is not yet clear, but it is attractive to speculate that unknown soluble serum factors which are associated with a low number of CTCs may suppress RANKL expression and/or alter processing of membrane bound RANKL into soluble RANKL. These studies now need to be confirmed in additional cohorts. Since an antibody to block RANKL (Denosumab) has already been approved for treatment of osteoporosis patients and treatment of skeletal related events in solid tumors [3, 7], our data in the presented prospective human cohorts further support the notion that the same antibody could potentially be used to prevent the development of breast cancer in high risk women. Advancement of our findings towards clinical application awaits prior validation in independent patient cohorts.

## MATERIALS AND METHODS

### UK collaborative trial of ovarian cancer screening (UKCTOCS)

The first group of subjects were participants in the UK Collaborative Trial of Ovarian Cancer Screening (UKCTOCS) [24, 25], a multi-centre randomized controlled trial of ovarian cancer screening that has recruited 202,638 postmenopausal women. The trial was set up at 13 National Health Services (NHS) trusts in England, Wales and Northern Ireland and is coordinated by the Gynecological Cancer Research Centre at the University College London (UCL). Women aged 50-74 were recruited through random invitation from age/sex registers of 27 participating Primary Care Trusts. At recruitment, written consent was obtained which included access to their medical records and use of their data/samples in future secondary studies. UKCTOCS was approved by the UK North West Multicentre Research Ethics Committees (North West MREC 00/8/34). Women were randomized in a 2:1:1 ratio to (1) a control group with no intervention (101 359 women), (2) a multimodal group with annual screening with CA125 (50 640 women) and (3) an ultrasound group with annual screening with ultrasound (50 639 women). Eligibility criteria included (a) age between 50 and 74 years and (b) postmenopausal status defined as > 12 months amenorrhea following a natural or surgical menopause, or > 12 months of hormone replacement therapy commenced for menopausal symptoms. The exclusion criteria were: (a) history of bilateral oophorectomy, (b) active non-ovarian malignancy (women with a past history of malignancy were eligible if they had no documented persistent or recurrent disease), (c) increased risk of ovarian cancer because of familial predisposition and (d) previous history of ovarian cancer. All women are followed through the national cancer registries and follow-up postal questionnaires. For confirmation of diagnosis, their treating physician was sent a questionnaire requesting information regarding their diagnosis (histopathology) and treatment. UKCTOCS was approved by the UK North West Multicentre Research Ethics Committees (North West MREC 00/8/34).

For the current study, eligible cases, who developed estrogen receptor positive invasive breast cancer after joining the trial being identified through the cancer registries or self-reporting on follow-up questionnaire, donated a serum sample between 5 and 24 months prior to diagnosis (N = 98). Out of these 98 women 58 women donated a sample between 12 and 24 months prior to diagnosis (median age at sample taken 60.49 years) and 40 between 5 and 12 months (median age at sample taken 64.84). Eligible controls were matched UKCTOCS participants who had no history of breast cancer at last follow-up (median time between sample collection and follow up is 3.24 years) and had donated serum samples on the same day and in the same clinic (N = 180) as cases (median age at sample taken 62.94 years). Clinicopathological data of the cases are provided in Table 1. Each breast cancer case was age-matched with two control women. Both cases and controls were not on hormone replacement therapy (HRT) at the time of sample collection. Blood samples were collected into Griener Bio one gel tubes (Cat no: 455071) at the centers, shipped overnight to the central laboratory and centrifuged at 2000 g for 10 minutes. The serum was removed from the cells within 56 hours of sample collection and was frozen using a two stage freezing process: 12 hours at −80°C and then placed in liquid nitrogen (vapor phase) -1 800°C. A semi-automated system aliquoted serum in 500 μl straws which were then heat sealed, bar coded, and stored in liquid nitrogen tanks. One straw was retrieved and the samples were only thawed before use. Ethical approval for this nested case control study was obtained from the Joint UCL/UCLH Committees on the Ethics of Human Research (REC reference: 06/Q0505/102).

### SUCCESS trial

Eligible patients were defined as women with histologically confirmed invasive breast cancer (stages pT1-4, pN0-3, M0) who had their tumors completely removed and agreed to participate in the randomized phase III SUCCESS A study. The main inclusion criterion was the indication for adjuvant chemotherapy defined by either positive axillary nodal status or node negative disease with additional factors associated with an increased risk for recurrence, i.e. pT ≥ 2, grade 3, age younger than 35 years or negative hormone receptor status. The SUCCESS A study compared the disease-free survival after randomization in patients treated with 3 cycles of Epirubicin (100 mg/m^2^)-Fluorouracil (500 mg/m^2^)-Cyclophosphamide (500 mg/m^2^, FEC) chemotherapy every three weeks (q3w) followed by 3 cycles of Docetaxel (100 mg/mg^2^, D) q3w *versus* 3 cycles of FEC q3w followed by 3 cycles of Gemcitabine (1,000mg/m^2^ d1,8)-Docetaxel (75 mg/m^2^, DG) q3w. After chemotherapy, patients were randomized to receive two (q 3 months x 24 months) *versus* five years of zoledronate (q 3 months x 24 months followed by q 6 months x 36 months). Women with hormone receptor positive disease received endocrine treatment. Premenopausal women received tamoxifen alone or in combination with goserelin for two years if they were younger than 40 years of age or became premenopausal within 6 months of chemotherapy. Hormone receptor positive postmenopausal patients were treated with tamoxifen for two years followed by anastrozole for three years. 2026 patients had a blood sample drawn after R0 resection of the primary tumor before the start of adjuvant chemotherapy. All blood samples were obtained after written informed consent. The study was approved by all responsible ethical boards and conducted in accordance with the Declaration of Helsinki. Ethics number: 076-05 (Ethical committee of the Ludwig Maximilian University of Munich). EudraCT Nr.: 2005-000490-21; Protocol ID: SUCCESS - Vorlage Nr.: 4030471.

### CTC analysis

CTCs [26, 27] were analyzed using the CellSearch System (Veridex, USA). Peripheral blood was drawn into three CellSave preservative tubes containing a cell stabilizing agent. The samples were shipped at room temperature to the central cancer immunological laboratory at the Women´s Hospital of the University of Munich Innenstadt and analyzed within 96 hours after arrival. Briefly, 30ml of blood was pooled and reduced to 7.5 ml by centrifugation of the sample for 10 minutes at 800 x g. Plasma was removed and dilution buffer added and this mixture was overlaid over 6 ml of Histopaque using a syringe with valve and tubing, before another centrifugation step for 10 minutes at 400 x g. Subsequently, 7.5 ml of this sample containing the buffy coat was processed automatically using a CellTracks AutoPrep System. The CellSearch Epithelial Cell Kit was used for CTC enrichment and enumeration. After immune-magnetic enrichment with an anti-Epcam antibody bound to magnetic beads, cells were labeled with fluorescent anti-cytokeratin (CK 8,18,19-phycoerythrin) and anti-CD45 antibodies (CD45-allophycocyan) to distinguish between epithelial cells and leukocytes. The fluorescent nucleic acid dye 4,6-diamidino-2-phenylindole dihydrochloride was used to detect viable cells. The identification and enumeration of CTCs were performed with the use of the CellTracks Analyzer II, a semi-automated fluorescence-based microscopy system that permits computer-generated reconstruction of cellular images. CTCs were defined as nucleated cells lacking CD45 and expressing cytokeratin. All positive samples were reviewed by two independent observers. For each CellSearch Epithelial Cell Kit, calculated for 15 patient samples, a test sample with a defined number of breast cancer cells was evaluated as a positive control.

### Bruneck cohort

Breast cancer patients in the Bruneck cohort were composed of patients with ER/PR negative breast cancer, ER/PR positive breast cancer, which had received tamoxifen but had stopped it when blood was withdrawn and a small group of ER/PR positive breast cancer, which still received tamoxifen when blood was withdrawn. There was no difference in RANKL/OPG changes among these groups suggesting that the observed changes occur independent of anti-hormone therapy. All data in females and males were corrected for age, sex, body mass index, menopausal state, smoking, alcohol consumption, social status, physical activity, diabetes mellitus, creatinine levels and medication such as glucocorticoids and, importantly, hormone replacement therapy. For recruitment details in the prospective population-based Bruneck Study see refs. [28, 29]. A total of 15 subjects with cancer at baseline were excluded (three of them had a breast cancer). Of the 894 subjects eligible, 748 remained free of cancer during the 15-year follow-up between 1990 and 2005, 19 developed breast cancer, 16 developed prostate cancers, and 89 non-breast cancers (all malignancies except for squamous-cell skin cancer). Cancer status was ascertained by the patient’s self-report and medical records. Tumors were obligatorily confirmed by histology. The situation in Bruneck is unique in that the hospital houses the only radiological facility and pathological laboratory in the whole region.

### Serum RANKL, OPG, and hormone measurements

Serum samples were immediately frozen and stored and transferred at -80°C before use. Matched case and control samples were handled identically and were assayed in random order. Laboratory personnel were unaware of the case-control status during all assays. Serum levels of progesterone were measured using a chemiluminescent immunoassay (with an Elecsys 2010 autoanalyzer, Roche Diagnostics). According to the manufacturer both intra- and inter-assay coefficients of variation were lower than 10% with detection limits of 0.095 nmol/l for progesterone. Serum levels of soluble RANKL and OPG were measured using a sandwich and competitive enzyme immunoassay (Biomedica, Vienna, Austria) as previously reported. According to the manufacturer, both the intra- and inter-assay coefficients of variation were lower than 10% with detection limits of 0.14 pmol/L for OPG and 0.08 pmol/L for soluble RANKL. Details on the measurement of OPG, free RANKL, and progesterone *via* ELISA have been reported for the Bruneck cohort [28, 29].

### Statistical analysis

For analysis of the Bruneck cohort, statistical calculations were performed using the SPSS 15.0 and BMDP software packages. Concentrations of OPG and RANKL, and the RANKL/OPG ratio were log_e_-transformed to approximate a normal distribution. Variable levels prior to and after cancer manifestation were compared with the paired *t*-test. Changes of variable levels among subjects with incident breast cancer, subjects with new-onset non-breast cancer and those who remain free of neoplastic disease were compared by means of general linear models (GLM) for repeated measurements. The same analyses were performed for male subjects. To account for the varying time intervals and to eliminate potential effects of ageing, all OPG and RANKL measurements were adjusted to baseline age of given individuals by linear regression techniques. Cox proportional hazard models were used to assess whether baseline levels of OPG and RANKL or the RANKL/OPG were independent risk predictors for the manifestation of breast, prostate, and non-breast/non-prostate cancer. Subjects who developed cancer were censored with respect to subsequent follow-up. Proportional hazard assumptions were confirmed by testing each variable with an interaction for time (Cox models with time-dependent covariates). Sensitivity analyses excluded subjects who received a cancer diagnosis within three years after baseline and yielded virtually identical results for all computations specified above. All reported P values are two-sided.

For the UKCTOCS studies and SUCCESS trial, statistical analyses were conducted with the use of R software, version 2.11.1. To compare univariate distributions standard boxplots were utilized with 0.25, 0.5 and 0.75 quantiles standing for box bottom, horizontal line and box top, and samples extremes for whiskers, to test difference between distributions Mann Whitney U test (Wilcoxon test) has been used. To stratify the risk for women with high progesterone Odds ratios and 95% confidence intervals were calculated through the conductance of logistic regression analyses for the categorical values. One tertile (1st for RANKL and 3rd otherwise) was referred as a reference value and Odds Ratios (ORs) of two other tertiles have been calculated with respect to the reference value. These ORs have been considered separately for cases with time to diagnosis more than 1 year and less, and accompanied with the corresponding confidence interval (CI) and P Value of the hypothesis that OR is different from one.
